# Development of Pistachio Shell-Based Bioadsorbents Through Pyrolysis for CO_2_ Capture and H_2_S Removal

**DOI:** 10.3390/molecules30071501

**Published:** 2025-03-27

**Authors:** Alejandro Márquez Negro, Verónica Martí, José María Sánchez-Hervás, Isabel Ortiz

**Affiliations:** 1 Unit for Sustainable Thermochemical Valorization, Energy Department, CIEMAT, 28040 Madrid, Spain; veronica.marti@ciemat.es (V.M.); josemaria.sanchez@ciemat.es (J.M.S.-H.); isabel.ortiz@ciemat.es (I.O.); 2 Department of Chemical Engineering and Materials, Faculty of Chemistry, Complutense University of Madrid (UCM), 28040 Madrid, Spain

**Keywords:** biochar, hydrogen sulfide, adsorption, waste valorization, carbon capture

## Abstract

The development of sustainable waste management for environmental remediation has highlighted the potential of biochar produced from agricultural wastes as an effective adsorbent for gas pollutant capture. This work focuses on the production and activation of biochar derived from pistachio shells for CO_2_ and H_2_S adsorption. Adsorbents were obtained by pyrolysis and subsequently activated through two methods: chemical activation with KOH and physical activation with CO_2_. Adsorption studies were conducted to evaluate the influence of these activation methods on textural properties and adsorption capacities. Chemical activation enhanced microporosity and increased the specific surface area (531 m^2^/g), resulting in a better performance, obtaining adsorption capacities of 87 mgCO_2_/g_adsorbent_ and 9.6 mgH_2_S/g_adsorbent_. Non-linear kinetic models were identified as the most suitable for fitting CO_2_ adsorption data, with the Avrami model presenting the best fit results. Dynamic H_2_S adsorption tests revealed the influence of moisture present in the adsorbent, favoring H_2_S dissociation and thus improving capture processes, especially when chemical activation biochar is employed. This enhancement is attributed to the greater development of active centers on its surface, including micropores and heterogeneous atoms introduced though impregnation.

## 1. Introduction

Pyrolysis is a thermochemical process that could valorize several residues from diverse nature, including lignocellulosic biomass, plastics, textiles, etc. [1]. During the process, the feedstock is decomposed in the absence of oxygen, generally in an inert atmosphere (N_2_), at moderate temperatures between 300 and 900 °C. As a result, three main products are obtained: a solid material or biochar, the liquid fraction or bio-oil, and non-condensable gases. Product distribution depends on several operational parameters, including residence time, heating rate, pyrolysis temperature, and feedstock nature. If biochar is the objective product, slow heating rates and long residence times are required [2]. As a result, a carbon-rich solid with high surface area and porosity is obtained. However, its physicochemical properties are highly influenced by process conditions. Biochar can be further treated, through physical or chemical activation, to improve its adsorption properties.

In physical activation, biochar is subjected to a thermal treatment at high temperatures, but, in this case, under an oxidizing atmosphere, usually of CO_2_ or steam [3]. This process favors oxidation reactions that enhance pore formation, develop the porous structure, and increase its surface area [3]. On the other hand, chemical activation requires a chemical agent, which can be acid or alkali and is impregnated into the biochar. Afterwards, it is thermally treated at lower temperatures, compared to physical activation, between 500 and 700 °C in an inert atmosphere. During this step, the chemical agent penetrates into the carbon matrix, promoting further degradation of the volatile matter through dehydration reactions [4]. As a result, the increase in biochar’s porosity is achieved, particularly enhancing its microporous structure and incorporating functional groups and heteroatoms on its surface [5].

Many authors, instead of performing a two-stage activation process—first pyrolysis and then activation—carry out the activation directly on the sample and then pyrolyze it, which simplifies the process [4]. However, the main drawback, often overlooked, is that during pyrolysis, not only biochar is produced, but so are bio-oil and non-condensable gases. Bio-oil can be upgraded and used as an alternative fuel or as a platform for value-added chemicals [6]. However, when an activating agent is added, the resulting bio-oil may contain traces of the activating agent or derivatives, leading to bio-oil contamination and reducing the overall efficiency of the process.

Agricultural wastes have received significant attention as feedstock for biochar production due to their abundance and low cost. Pistachio was one of the fastest growing crops across the last decade in Spain. In 2022, the cultivated area of this crop exceeded 70,000 ha, representing a 3000% increase over the past decade [7]. This exponential growth is driven by high international and domestic demand, combined with favorable continental climates in certain regions of the country. The main residue generated in pistachio cultivation is the shell. This residue presents high carbon content, along with its high availability and low cost, which make it an ideal feedstock for biochar production [8].

In recent years, the use of biochar derived from biomass waste as a sorbent for aqueous pollutant removal has gained scientific interest. Kurunogly and Demi [9] investigated the potential of using biochar from pistachio shell (PS), providing a review of the developments made regarding adsorbing metals [10], dyes [11], and antibiotics [11] in aqueous solutions. However, few studies have focused on its application for the removal of atmospheric contaminants, especially H_2_S, which is present in numerous industrial processes. For instance, in anaerobic digestion, biogas must undergo an upgrading process prior in order to be suitable for its application. The use of bioadsorbents produced from waste would not only result in cost reductions but would also promote a circular economy, as the waste from one process serves as a raw material for another. In addition, biochar presents an alkaline nature, which can also enhance the capturing of acidic gases like CO_2_ and H_2_S [12]. Villardon et al. synthesized activated carbons from pistachio shell using single-step physical activation with steam as the activating agent and measured the CO_2_ adsorption capacity, achieving 2.81 mmolCO_2_/g_adsorbent_ at 10 bar [13]. However, these high-pressure conditions are not optimal for CO_2_ capture from biogas, as this stream is typically at atmospheric pressure. Despite the extensive studies on CO_2_ capture via biomass-derived biochar adsorption, there is a limited number of studies that examine both CO_2_ and H_2_S adsorption capacities, and, to our knowledge, no studies have investigated the use of pistachio shell biochar (PSB) for this purpose.

Therefore, the aim of this study is to develop bioadsorbents from pistachio shell for CO_2_ and H_2_S capture, combining environmental and waste management benefits. The novelty of this study lies in the exploration of adsorption capacities for CO_2_ and H_2_S using bioadsorbents prepared by two different activation methods from biochar. Adsorbents were prepared through two activation methods: chemical activation with KOH solution and physical activation using CO_2_ as the activating agent. CO_2_ adsorption capacity was studied through static adsorption tests, where linear and non-linear kinetic models were employed to determine the adsorption kinetics parameters. For H_2_S capture, dynamic adsorption experiments were conducted, investigating the influence of moisture present in the adsorbents. The findings of this research will offer significant insights into the adsorption processes of CO_2_ and H_2_S on activated biochars.

## 2. Results and Discussion

### 2.1. Adsorbents Characterization

#### 2.1.1. Elemental Analysis

Table 1 presents the elemental analysis of the different adsorbents studied. The results demonstrate that pyrolysis leads to an increase in the carbon content, from 46% in PS to 88% in PSB, reducing the oxygen content and also the hydrogen content. The activation methods also influence the carbon content of the adsorbent. While the chemical activation of pistachio shell biochar (PSB-CA) results in a decrease to 78%, physical activation (PSB-PA) leads to an increase in carbon, reaching up to 95% carbon in the adsorbent.

As temperature increases, the aromatization and hydrophobicity of the biochar also increase. Due to the removal of the polar functional groups from the surface through dehydration and decarboxylation reactions, a reduction in the O/C ratio is observed in the case of PSB-PA [14,15]. However, in PSB-CA, a slight increase in the O/C ratio is observed due to impregnation with KOH, which introduces oxygenated groups onto the surface.

All biochar-based adsorbents exhibit alkalinity, which could enhance their interaction with acid molecules such as CO_2_ or H_2_S. Regarding the yields, the char yield from PSB is 23%, with bio-oil forming a predominant fraction of the pyrolysis process. In addition, activation processes do not significantly reduce the overall solid yield, as they only result in a decrease of 3% for physical activation and 0.5% for chemical activation.

#### 2.1.2. Thermal Stability

**Figure 1:**
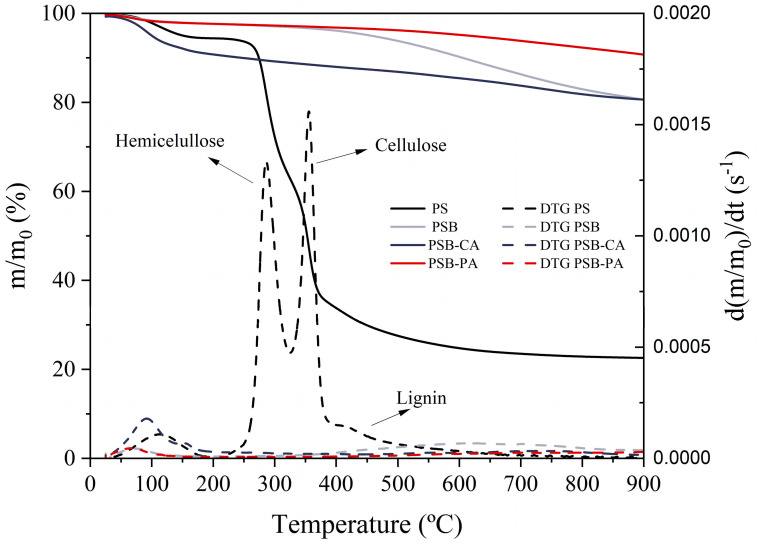
Thermogravimetric analysis of the adsorbents at a heating rate of 10 °C/min under pyrolysis conditions; TG (solid line) and DTG (dotted line).

Figure 1 depicts the thermal stability of the adsorbents produced. The solid line shows the weight loss as a function of temperature (TG), while the DTG curve (dotted lines) represents the decomposition rate, which allows for the identification of the different degradation stages. In PS pyrolysis, four decomposition stages are revealed. First, the moisture loss occurs between 80 and 150 °C, with a weight loss of 5.5%. Subsequently, at 250 °C, the decomposition of hemicellulose begins, with the highest decomposition rate at 290 °C. This is followed by the degradation of cellulose (300–400 °C), reaching a maximum decomposition rate at 355 °C [16]. Finally, the decomposition of lignin is observed, with a small shoulder around 400 °C. However, lignin degradation occurs over a wide temperature range (250–550 °C), indicating that lignin degradation also takes place during the decomposition of cellulose and hemicellulose [17]. The adsorbents produced from PS exhibit high thermal stability. All samples present an initial stage of moisture and physisorbed compound loss at 80–120 °C, with the most significant mass loss observed for PSB-CA (≈8%). This result aligns with the findings of Fu et al., who attributed this phenomenon to the hygroscopic property of alkali potassium [18]. A second step follows, characterized by the volatilization of the char. The PSB sample shows a marked loss beginning at 450 °C, as this was the final pyrolysis temperature, and thus, the sample has not been exposed to higher temperatures.

#### 2.1.3. Surface Area

Table 2 presents the results of the surface characterization of the adsorbents synthesized. PS presents a minimal porous structure, without any pore volume or micropores. The pyrolysis of the PS enhances its porosity and increases the Brunauer–Emmett–Teller (BET) surface area to 293 m^2^/g. A significant development of microporosity (V_p micro_) is observed, reaching 0.14 cm^3^/g. Both activation methods further increase the BET surface area compared to PSB. PSB-CA presents the highest BET surface area (531 m^2^/g) and the greatest microporosity, with a micropore volume of 0.21 cm^3^/g (90% of the total volume). On the other hand, PSB-PA presents a slight increase in mesopore volume, with 53% of the pores, demonstrating the capacity of CO_2_ to enhance the mesoporosity of the adsorbents in physical activation [19]. The mesopore volume increment could be attributed to higher activation temperature, which promotes volatilization reactions, leading to micropore collapse and the formation of mesopores, thus following the same tendency reported by Hong et al. [20].

**Figure 2:**
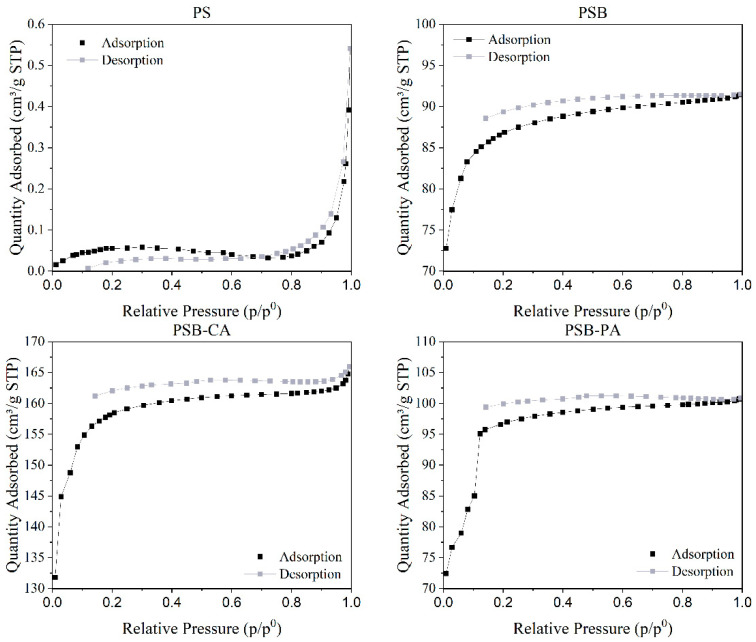
N_2_ adsorption–desorption isotherms of the synthesized adsorbents.

Additionally, N_2_ adsorption–desorption isotherms of the adsorbents are presented in Figure 2. According to the IUPAC classification, the N_2_ adsorption–desorption isotherm of the PS could be classified as Type III, where there is a lack of adsorption due to the weak adsorbate–adsorbent interaction. In contrast, the isotherms for PSB, PSB-CA, and PSB-PA can be classified as Type I-b with an H4 hysteresis loop [21]. It has been observed that none of the adsorption isotherms close with the desorption isotherms at low pressures. This phenomenon, known as low-pressure hysteresis, is attributed to the strong presence of micropores [22].

#### 2.1.4. Morphology and EDS Surface Analysis

**Figure 3:**
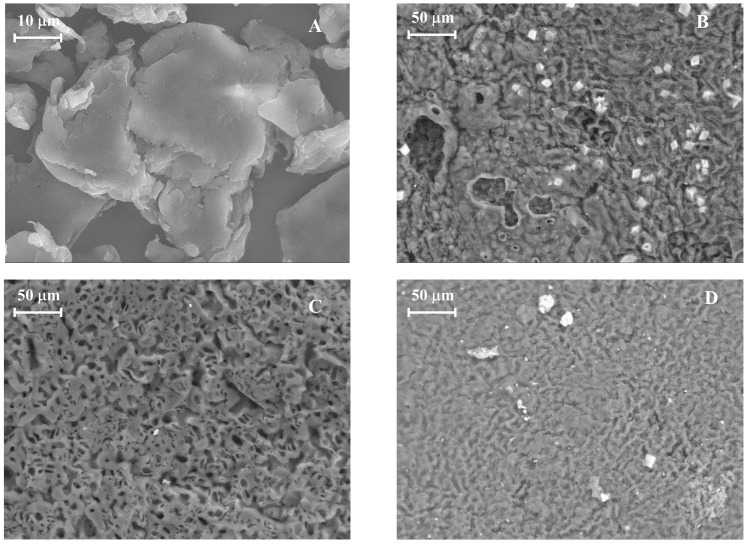
SEM images of PS (**A**), PSB (**B**), PSB-CA (**C**), and PSB-PA (**D**).

The effects of pyrolysis and activation processes on the adsorbent structure and porosity are observed through morphological and structural analysis using SEM-EDS (Figure 3). PS shows almost no porosity, presenting a smooth surface. After pyrolysis (PSB), the sample develops a more heterogeneous surface with grooves and channels. Physical activation further enhances the development of these grooves; however, it does not fully develop micropores, as evidenced in Table 2. This is achieved with chemical activation, where the development of a honeycomb-like structure with more pronounced pores is observed. Regarding the EDS analysis, it shows that pyrolysis reduces the oxygen content of the sample, increasing the carbon matrix. On the other hand, chemical activation with KOH (PSB-CA) introduces potassium onto the surface, as seen in the EDS spectrum (see Figure S1 in the Supplementary Materials). In addition, the adsorbents exhibit small crystals on the surface corresponding to calcifications (CaCO_3_) related with the main element present in PS ashes [23].

### 2.2. CO_2_ Adsorption Studies

**Figure 4:**
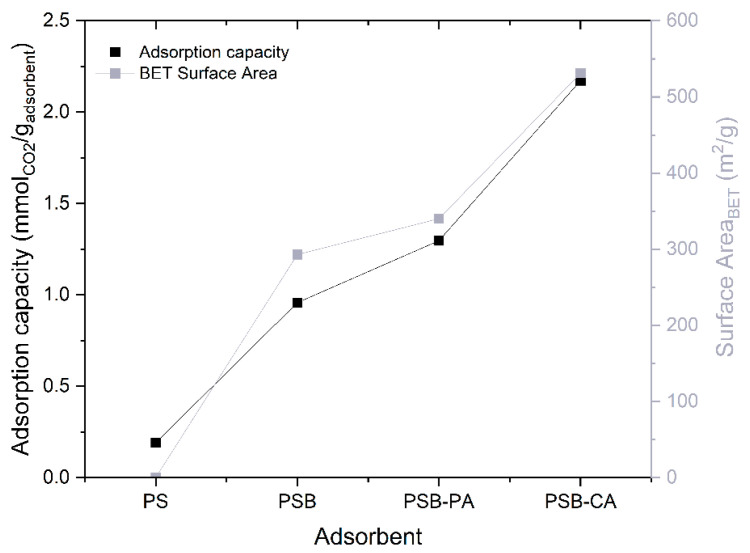
Correlation between CO_2_ adsorption capacity and BET surface area for the adsorbents.

PSB-CA exhibits the highest CO_2_ capture rate (2.17 mmolCO_2_/g_adsorbent_), indicating that KOH activation significantly enhances the adsorption capacity of the biochar. It is followed by PSB-PA, PSB, and PS with 1.30, 0.96, and 0.19 mmolCO_2_/g_adsorbent_, respectively. PS exhibits almost no adsorption capacity and therefore slower adsorption kinetic. Figure 4 illustrates the relationship between the specific surface area and the capture capacity of the adsorbents, clearly demonstrating that a higher surface area correlates with greater adsorption capacity. Surface area and microporosity play a significant role in physisorption, which is the primary mechanism contributing to the CO_2_ capture capacity of the biochar-based adsorbents [24]. In addition, the alkalinity of the adsorbent plays a major role in CO_2_ uptake. The presence of basic elements, such as K in the case of PSB-CA, enhances CO_2_ capture through chemical sorption by forming salts like K_2_CO_3_, as demonstrated by Xu et al. [25]. The adsorption results of PSB-CA are highly competitive compared to other adsorbents (0.7 to 5.4 mmolCO_2_/g_adsorbent_), such as metal–organic frameworks (MOFs), zeolites, activated carbons, or porous silica [26,27]. This competitiveness is not only due to its adsorption capacity falling within the expected range, but also because its synthesis follows a waste-to-resource strategy, contributing to environmental sustainability [28].

Table 3 compares the results of CO_2_ uptake obtained in this study with previous biomass-based sorbents derived from studies similar to this one. The CO_2_ adsorption capacity of biomass-derived activated carbon is closely related to its precursors and activating method. From Table 3, it is observed that the chemical activation of biochar generally presents better CO_2_ capture performance than physical activation, which aligns with the result obtained in this study. This is to be attributed to the development of the microporous surface through KOH activation, enhancing CO_2_ adsorption through Van der Walls forces, suggesting that the process primarily relies on physical adsorption [29]. Bamboo sawdust and wood pellets exhibited lower CO_2_ adsorption capacities, but this can be explained by the fact that those experiments were conducted under a 15% CO_2_ flow instead of pure CO_2_, as employed for the other studies.

On the other hand, chicken manure-activated biochar showed low SSA (22 m^2^/g) but a surprisingly high adsorption capacity with the other adsorbents. This phenomenon could be attributed to the presence of nitrogen functional groups on the biochar surface, which facilitates CO_2_ chemisorption, as reported by Jung et al. [4]. The ammoxidation of biochar introduces active adsorption sites, especially pyrrolic nitrogen, which reacts with the CO_2,_ leading to the highest adsorption capacity [30]. This effect is evident in spent coffee ground biochar activated via ammoxidation and KOH, where nitrogen functional groups are first doped on the adsorbent surface, followed by the development of porosity through KOH activation.

**Table 3:** CO_2_ adsorption capacities of various biomass-derived biochar reported in the literature to be determined through TGA.

Feedstock	Pyrolysis Temperature (°C)	Activation Method	SSA (m^2^ g^−1^)	Adsorption Conditions	CO_2_ Adsorption Capacity (mmol g^−1^)	REF
Pistachio shell	450	None	293	30 °C and 1 bar	0.96	This work
Almond shell	600	None	21	25 °C and 1 bar	1.59	[31]
Sawdust	500	None	22	25 °C and 1 bar	0.98	[32]
Coconut shell	1000	None	1250	25 °C and 1 bar	0.53	[33]
Pistachio shell	450	CO_2_ (700 °C for 1 h)	340	30 °C and 1 bar	1.3	This work
Wood pellet	1000	CO_2_ (550 °C for 1 h)	287	25 °C and 1 bar	0.26	[33]
Almond shell	600	CO_2_ (750 °C for 2 h)	1090	25 °C and 1 bar	2.7	[34]
Pistachio shell	450	KOH (600 °C for 1 h)	531	30 °C and 1 bar	2.17	This work
Bamboo Sawdust	1200	KOH (550 ° for 1 h)	526	25 °C and 1 bar	0.69	[33]
Chicken manure	400	KOH (700 °C for 1 h)	22	25 °C and 1 bar	1.95	[35]
Coffee grounds	400	Ammoxidation and KOH (600 °C for 1 h)	990	35 °C and 1 bar	2.67	[30]

#### 2.2.1. Effect of CO_2_ Concentration

**Figure 5:**
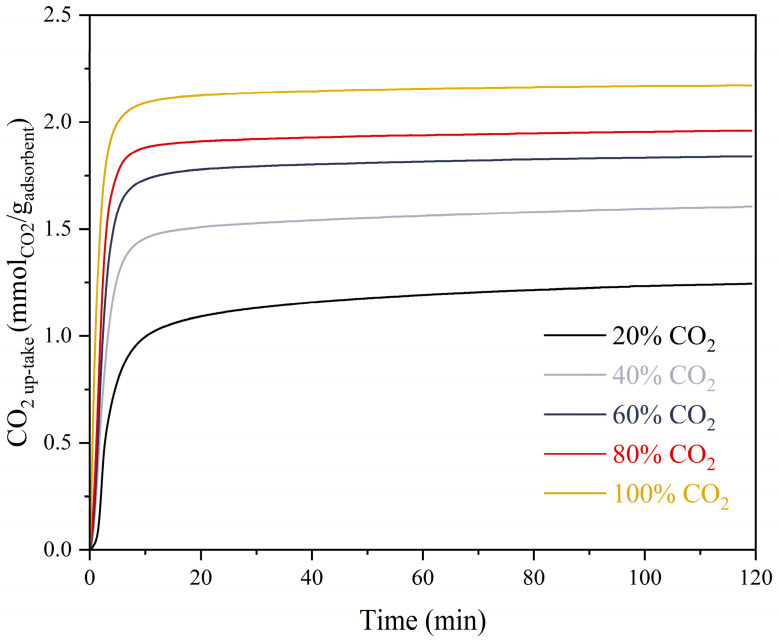
PSB-CA adsorption curves for different concentrations of CO_2_ (20, 40, 60, 80, and 100% *v*/*v*).

PSB-CA was exposed to varying CO_2_ concentrations in the feed flow, while maintaining a total flow rate of 50 mL/min. CO_2_ adsorption curves and the adsorption capacity were studied for CO_2_ concentrations of 20%, 40%, 60%, 80%, and 100% (*v*/*v* CO_2_/N_2_). The adsorption capacities at 30 °C and 1 atm are depicted in Figure 5. It can be observed that the adsorption capacity increases with higher CO_2_ concentrations. The adsorption capacity of PSB-CA at 20% *v*/*v* CO_2_ was 1.24 mmol CO_2_/g_adsorbent_, which increased to 1.6, 1.83, 1.96, and 2.17 mmolCO_2_/g_adsorbent_ for CO_2_ concentrations of 40%, 60%, 80%, and 100% *v*/*v*, respectively. This increase is attributed to the higher availability of adsorbates, which interact with the active sites. Additionally, at higher concentrations, multilayer adsorption effects occur, increasing the CO_2_ capacity of the adsorbent. In addition, when CO_2_ concentration increases, a decrease in the time required to reach equilibrium can be observed. At 20% CO_2_ *v*/*v*, equilibrium was reached after 15 min, whereas at 100% CO_2_ *v*/*v,* it was achieved in 6 min.

#### 2.2.2. Regeneration Performance

**Figure 6:**
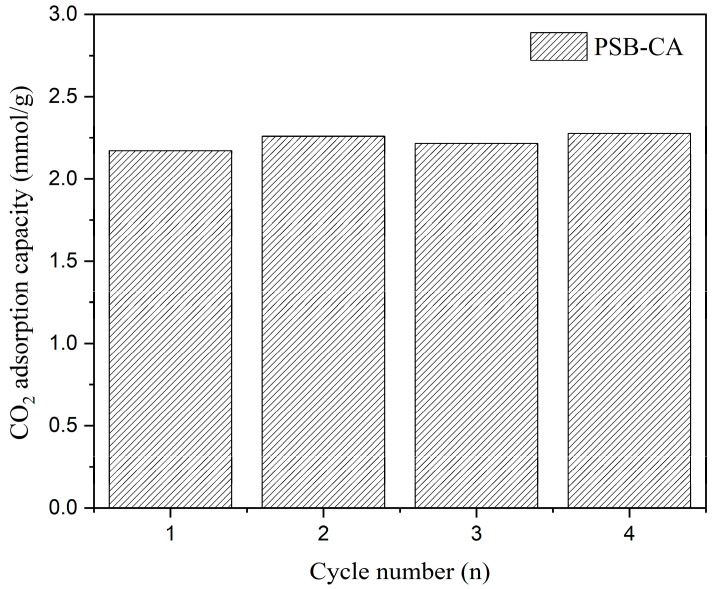
CO_2_ adsorption capacity of PSB-CA during cyclic adsorption–desorption.

In industry, not only the adsorption capacity and selectivity of the sorbent are important, but also is its regeneration capacity in cyclic adsorption–desorption processes [36]. Therefore, four adsorption–desorption cycles were performed to determine the regeneration capability PSB-CA using a temperature swing adsorption process. The adsorption and desorption temperatures were set at 30 °C and 120 °C, respectively. During adsorption, the flow was CO_2_, while in the desorption stage, it was switched to N_2_. The results of this study are presented in Figure 6, which shows that after four cycles, the adsorption capacity remains stable at 2.2 mmolCO_2_/g_adsorbent_. Thus, its repeated use did not affect CO_2_ sorption performance, indicating its high stability as CO_2_ bioadsorbent.

#### 2.2.3. CO_2_ Adsorption Kinetics

Adsorption kinetics helps to determine the time needed to reach equilibrium, enabling more efficient process design [37]. It also helps to identify whether physical or chemical adsorption dominates the process, which is crucial for enhancing material performance [38]. Traditionally, kinetic parameter determination has been performed through the linearization of kinetic models. The linearization methods are based on approximations, which can lead to uncertainty, ultimately resulting in inaccuracies [39]. However, recent advancements in software development enable the adjustment of experimental values using non-linear fitting. Despite these advancements, most studies are still focused on linear fitting. Therefore, in this study, we compare both approaches using three kinetic models: the pseudo-first order (PFO), pseudo-second order (PSO), and Avrami model. The equations for each model are presented in the Materials and Methods Section: Equations (2)–(4) correspond to the PFO, PSO, and Avrami models, respectively.

**Figure 7:**
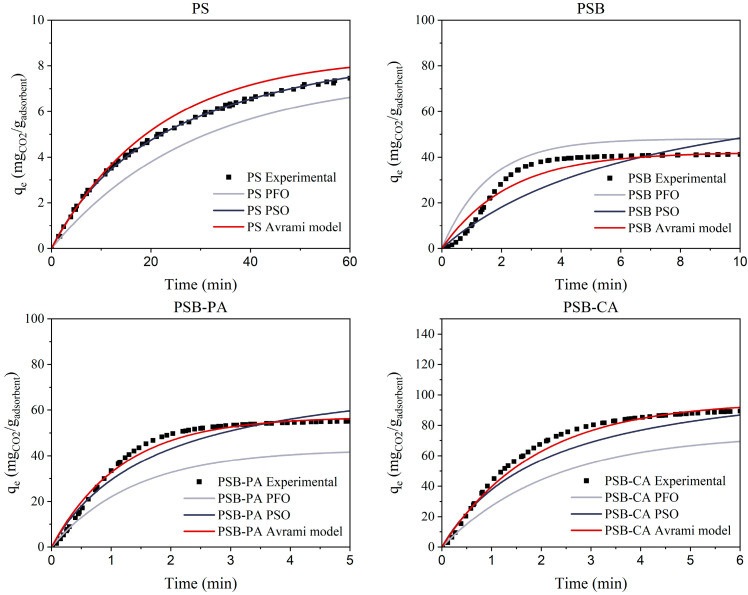
Adsorption curves of kinetic models fitted by linear fitting.

**Figure 8:**
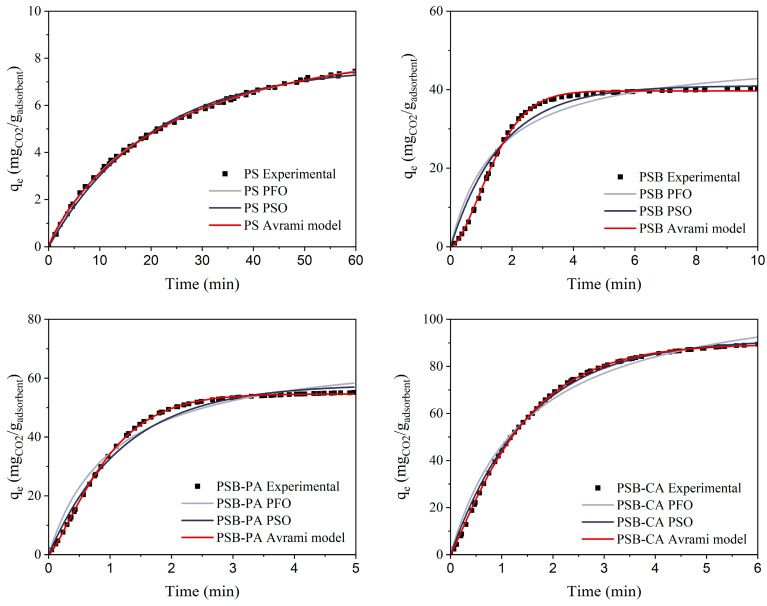
Adsorption curves of kinetic models fitted by non-linear fitting.

Figure 7 depicts the linear fit of each adsorbent (PSB, PSB-PA, and PSB-CA) to the three kinetic models described previously. Table 4 presents the kinetic parameters calculated for each adsorbent and linear fitting kinetic model. Additionally, Figure 8 and Table 5 display the kinetic model curves for non-linear fitting and its adsorption kinetic parameters. In linear fitting models, important discrepancies are observed between kinetic models and experimental data. The PFO and Avrami models show a greater R^2^ than 0.9, indicating that CO_2_ adsorption on the pistachio shell-derived biochars could follow these models. On the other hand, PSO significantly deviates from the experimental values, resulting in low R^2^ values. Another approach to assess if a model fits the experimental data is comparing the maximum adsorption capacity. In this case, the PSO model overestimates the adsorption capacity of each adsorbent by up to 50%, as shown in the PSB results. Meanwhile, for the PFO model, the largest deviation in q_e_ occurs with PSB-CA, where the theoretical value is 62 mgCO_2_/g_adsorbent_, compared to the experimental value of 87 mgCO_2_/g_adsorbent_.

On the other hand, non-linear fitting provides greater accuracy, demonstrating that this technique is much more reliable and offers a deeper knowledge of the adsorption phenomena, as seen in Table 5 and Figure 8. R^2^ values greater than 0.9 are obtained for all the models, with q_e_ values closer to experimental values. Among the models, it is concluded that the Avrami model most accurately represents the experimental CO_2_ capture values. The parameters obtained from the Avrami equation provide valuable insights into the interaction of CO_2_ and the adsorbent’s surface and pore structure [40]. For PS, the n_Av_ value is significantly less than 1, indicating a slow adsorption process, mainly due to the limited number of available adsorption sites on the material. After pyrolysis, the number of adsorption sites increases, as reflected in a higher n_Av_ value for the PSB. In physical and chemical activation, the n_Av_ values decrease to slightly above 1, suggesting a more complex CO_2_ capture mechanism in these adsorbents. This suggests the formation of multiple layers of adsorbed CO_2_, leading to progressive adsorption within the pores, primarily due to the microporous structure of the adsorbents, especially for PSB-CA [41,42].

However, determining only the R^2^ value is not enough to determine if a model explains the experimental data [39]. Figure S2 in the Supplementary Material depicts normal probability plots of the adsorbents for the non-linear models studied, and Table S2 presents the RSME of the models. The results of this analysis allow us to determine if the model follows a normal distribution. It can be observed that the data obtained through the Avrami model align along the corresponding straight line, approximating to a normal distribution. In contrast, the PFO and PSO models deviate from the line, showing a fluctuating pattern. This trend occurs when there is a positive correlation between the error term and time; thus, these models must be excluded. In addition, RMSE analysis shows that non-linear fitting models match better with the experimental values. Among them, the Avrami model provides the best results for the studied adsorbents. Although the Avrami model was identified as the best fit for the kinetic curves, additional insights into the governing sorption mechanism of CO_2_ on biochar using adsorption isotherms could further enhance the understanding of the adsorption process.

### 2.3. Dynamic H_2_S Adsorption

**Figure 9:**
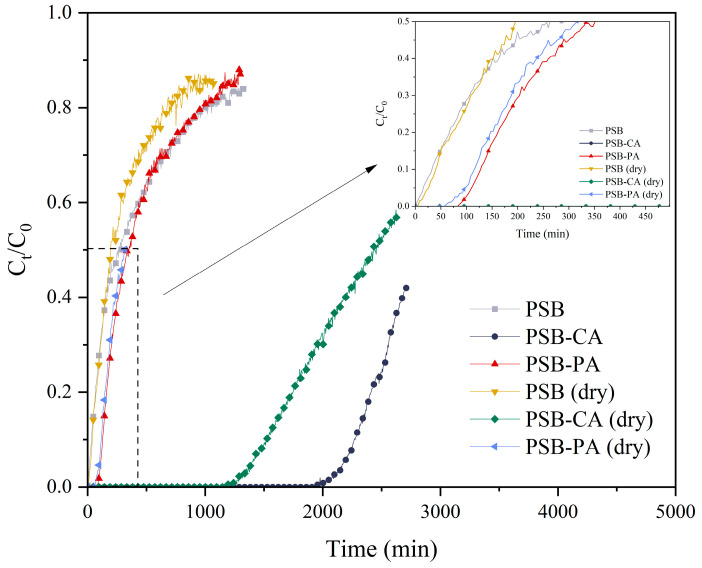
H_2_S breakthrough results for the biosorbents studied, with intrinsic moisture and dry conditions. (Adsorption: 30 °C, 100 ppm H_2_S, balance N_2_, GHSV = 1000 h^−1^, 1 bar).

Dynamic H_2_S capture experiments were conducted for the adsorbents derived from PS, with the exception of PS, which showed minimal CO_2_ adsorption capacity and was assumed to have zero H_2_S adsorption. In this work, we studied the moisture content effect of the biochar. For this purpose, tests were performed using the adsorbent with its original moisture content and after a drying pretreatment. The adsorbent’s breakthrough capacity was defined as the time when the H_2_S concentration reached 10 ppmv. Figure 9 presents the breakthrough curves for H_2_S capture. Similarly to CO_2_ capture results, chemically activated biochar (PSB-CA) demonstrated the best performance, with an adsorption capacity of 9.6 mgH_2_S/g_adsorbent_ and a breakthrough time of 2260 min. PSB-CA effectively removes H_2_S, leaving no detectable levels (<1 ppm) in the outlet gas before breakthrough occurred. In contrast, PSB and PSB-PA have significant lower H_2_S adsorption capacities, with values of 0.14 mgH_2_S/g_adsorbent_ and 0.58 mgH_2_S/g_adsorbent_, respectively. Some authors attribute H_2_S capture capacity fundamentally to the adsorbent’s higher surface area. However, this hypothesis may not fully explain the observed differences between the sorbents, as the variation in adsorption capacity is significantly higher than the difference in surface area. Furthermore, the adsorption capacity would be improved if the sorbent was subjected to a previous drying process, where moisture and physisorbed molecules would be removed, allowing H_2_S adsorption in the active sites. The results show that the surface area trend in adsorption capacity is consistent. However, in this case, their adsorption capacity decreases when the adsorbent undergoes dry pretreatment. PSB-CA shows a 33% capacity reduction, achieving an adsorption capacity of 6.4 mgH_2_S/g_adsorbent_, whereas PSB-PA present a capacity decrease to 0.52 mgH_2_S/g_adsorbent_, representing a 10% loss in H_2_S adsorption capacity.

The presence of moisture has a significant impact on H_2_S removal at low temperatures [43]. The TGA results show that PS and PSB-PA adsorbents exhibit very low moisture content, with only a 2% mass loss between 80 and 120 °C. The biochar surface from biomass is hydrophobic due to the aromatization of carbon chains during pyrolysis, which limits interactions with water [43]. Physical activation with CO_2_ does not enhance this interaction, as it does not add surface functional groups that increase the affinity for water [44]. In contrast, PSB-CA presents a more pronounced mass loss of 8% within the same temperature range, resulting in a higher moisture content and higher H_2_S adsorption capacity.

Choudhury and Lansing studied the effect of Fe-impregnation on different biochars derived from biomass. They reported H_2_S adsorption capacities of 0.5 mgH_2_S/g_adsorbent_ for corn stover biochar and 2 mgH_2_S/g_adsorbent_ for maple biochar; after Fe impregnation, the capacities increased to 1.5 and 15.2 mgH_2_S/g_adsorbent_, respectively [45]. Adib et al. concluded that a higher pH of H_2_S than pK_a_ (7.2) is necessary to allow for the dissociation of H_2_S on the water film, but the presence of metal oxides increases the process efficiency due to the catalytic oxidation reaction which produces elemental sulfur or sulfate [46]. The results of our study underscore the importance of enhancing the biochar-specific surface area and hydrophilic properties without reducing its pH. Therefore, PSB-CA biochar could be further employed as support for metal doping to improve desulfuration adsorption efficiency.

**Figure 10:**
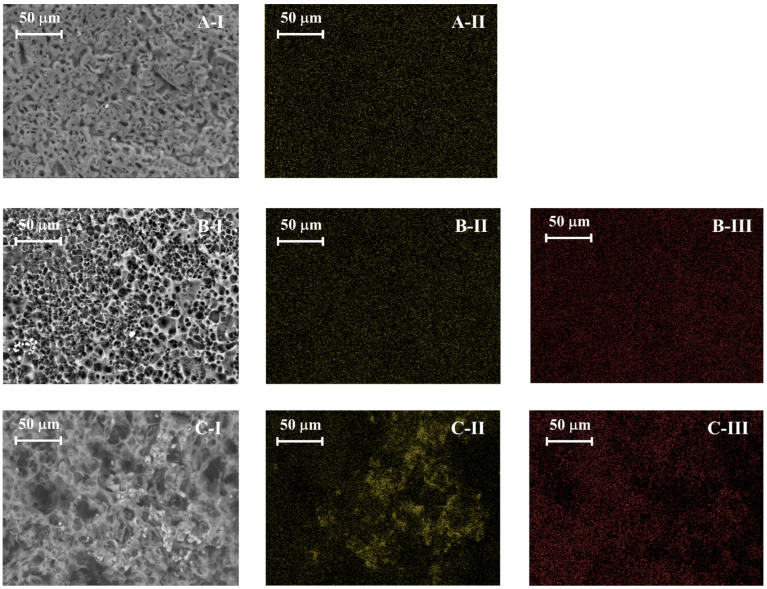
SEM and EDS mapping of fresh PSB-CA (**A-1**), PSB-CA after H_2_S adsorption (**B-1**), and PSB-CA after adsorption under dry conditions (**C-1**). The potassium distribution is color yellow (**A-II**, **B-II**, and **C-II**), while the sulfur content is in red (**B-II**,**C-III**).

In Figure 10, results from the SEM-EDS analysis for PSB-CA before and after H_2_S adsorption are presented. The surface morphology remains the same, as the adsorbents have not been subjected to a thermal process. EDS mapping demonstrates the presence of sulfur adhered to the adsorbent surface (highlighted in red). Sulfur adsorption occurs homogenously on the surface and does not directly take place on the potassium clusters. The concentration of sulfur present on the surface of the adsorbent obtained in SEM-EDS analysis is presented in Table S1. In addition, the SEM-EDS mapping distribution results of the other adsorbents (PSB and PSB-PA) are presented in Figures S3 and S4 in the Supplementary Materials, respectively. The detection of sulfur elements in SEM-EDS analysis confirms that the H_2_S adsorption of the activated biochar involves a chemisorption process, where sulfur is effectively fixed onto the carbon structure, as, if the process involved only physisorption, H_2_S would be desorbed under vacuum conditions.

**Figure 11:**
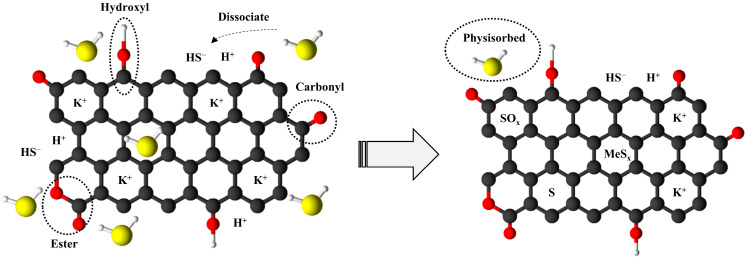
H_2_S adsorption mechanism on the activated biochar.

Several authors have reported that H_2_S adsorption on char involves a complex mechanism that significantly depends on the chemical properties of the char and the adsorption conditions [47]. Most of the studies conduct adsorption in the presence of oxygen at low concentrations, which promotes the oxidation of H_2_S. However, H_2_S gas streams do not always contain O_2_, as is the case with biogas streams and in this study. Yan et al. demonstrated the influence of a thin water layer in the microporous structure on H_2_S oxidation [43]. Figure 11 presents a schematic diagram of the proposed mechanism. The mechanism begins with H_2_S diffusion into the biochar, where they interact with the water layer, leading to the dissociation of H_2_S into HS^-^.

Thereafter, and according to Hervy et al., two reaction pathways could occur: (1) The direct reaction of HS^-^ with the metal oxides present in the adsorbent produces metal sulfides, which can afterwards be oxidized by the organic species present in the biochar, resulting in sulfates production [48]. (2) The present of oxygen-containing functional groups provided by the chemical activation could react with the H_2_S through a substitution reaction [49]. Furthermore, some H_2_S could also be physisorbed in the micropores and mesopores or dissociated in the water layer.

These results demonstrate the potential of the developed biochar for CO_2_ and H_2_S capture, unlike most previous studies that focus on only one of these gases. Using pistachio shell, a widely available and low-cost residue, the proposed valorization process helps to reduce waste and promotes environmental sustainability, making it a promising alternative for gas purification. However, further in situ operational studies are needed to better understand the adsorption mechanisms of CO_2_ and H_2_S in the activated biochars. To conclude, although PSB-CA yields higher adsorption capacity for both CO_2_ and H_2_S, the activation process requires higher chemical and energy cost, difficulties in chemical recovery, potential environmental risks, and the corrosion of the reactor and piping due to the strong alkali solution [5]. Therefore, a life cycle analysis to identify the environmental impact of each step and a techno-economic analysis to evaluate its economic feasibility could provide valuable insights for its optimization and implementation.

## 3. Materials and Methods

### 3.1. Materials

The feedstock studied for biochar-based adsorbent synthesis was Sirora pistachio shell (*Pistacia vera*), obtained from a plantation in the community of Madrid, Spain. The pistachio shells (PSs) were washed to remove any remaining ash or soil residue. After washing, the PSs were dried in an oven for 24 h at 100 °C. Once dry, the solid was grounded in a laboratory mill (Retsch SM2000, Düsseldorf, Germany) and sieved to a particle size of 0.5 to 2 mm.

### 3.2. Preparation of the Biochar-Based Adsorbents

The PSs are placed in a fixed-bed reactor and pyrolyzed at 450 °C for 15 min at a heating rate of 20 °C/min. N_2_ is used as the carrier gas at a flow rate of 100 mL/min. The pyrolysis takes place in a fixed-bed reactor with a diameter of 2.18 cm and a height of 38.5 cm made of stainless steel 316. This reactor is placed in a vertical furnace with a maximum temperature of 1200 °C. The reactor temperature is controlled using two thermocouples, one placed on the reactor wall and the other in contact with the sample. The condensate gases produced during pyrolysis are collected in two impingers placed in a cooling bath at 5 °C.

Afterwards, the pistachio shell biochar (PSB) obtained is activated using two different methods. The first method is a physical activation, where the PSB is placed into the same fixed-bed reactor described before, heated at a rate of 20 °C/min to 700 °C, and maintained for 1 h. The reaction atmosphere consists of an equal flow of N_2_ and CO_2_ at 50 mL/min. The activated biochar is collected after cooling with a 100 mL/min N_2_ flow. On the other hand, chemical activation involves impregnating PSB with KOH at a mass ratio 2:1. The impregnation ratio was calculated as the ratio of the weight of the biochar to the weight of KOH in a 1 M solution. To ensure the complete reaction occurred between PSB and KOH, the mixture was stirred constantly at 700 rpm at room temperature for 1 h. Then, the sample is dried at 100 °C overnight, activated at 600 °C in a fixed-bed reactor under N_2_ flow (100 mL/min) at a heating rate of 20 °C/min, and held for 1 h. Subsequently, the activated biochar is washed with deionized water until the pH is constant and then dried at 110 °C for 24 h. Physical- and chemical-activated biochars are referred to as PSB-PA and PSB-CA, respectively.

### 3.3. Adsorbents Characterization

Biochar-based adsorbents were characterized by elemental analysis following the UNE-EN standards (EN 15104, EN 15289, EN 15407, and EN 15408). The thermal stability of all the adsorbents was determined in a TGA2 system (Mettler Toledo Corporation, Greifensee, Switzerland). In a typical thermogravimetric analysis (TGA), 10 mg of each adsorbent were heated under flowing N_2_ (50 mL/min) from 30 to 900 °C at a heating rate of 10 °C/min. The biosorbent surface area was obtained after applying the Brunauer–Emmet–Teller (BET) model results obtained from N_2_ adsorption–desorption isotherms at 77 K in a Micromeritics ASAP 2020 device. On the other hand, the micropore volume (V_p micro_) and mesopore volume (V_p meso_) were calculated using the t-plot method; the total pore volume (V_T_) was determined considering the N_2_ volume adsorbed at P/P_0_ = 0.95; D_av_ was calculated using the Barrett–Joyner–Halenda method (BJH); and the porosity and the total intrusion volume were determined using mercury intrusion porosity in a Micromeritics AutoPore Series IV 9500. The surface morphology of the adsorbents was examined using a Scanning Electron Microscope (SEM) Zeiss EVO LS15, equipped with Energy Dispersive Spectroscopy (EDS) analysis (Oxford Inca Energy 350). Finally, pH was determined in a pH-meter (GLP 21, Crison), mixing the biochar-based adsorbent with deionized water in 10:1 water–biochar (mL:g) ratio for 1 h.

### 3.4. Adsorption Procedures

#### 3.4.1. CO_2_ Adsorbent Procedure

CO_2_ adsorption studies were performed using thermogravimetric analysis (Mettler Toledo TGA2 system). First, samples (10–20 mg) were degassed at 125 °C with a N_2_ flow of 50 mL/min for 2 h to remove moisture and physisorbed molecules. Subsequently, the samples were cooled to 30 °C under the same N_2_ flow. The CO_2_ adsorption capacity of the adsorbents was measured by changing the inlet gas to CO_2_ (50 mL/min) for 2 h or until a constant mass was observed. The increase in mass observed for the dried sample allowed for the determination of the CO_2_ adsorption capacity of the adsorbent, as seen in Equation (1).

(1) $$q_{e}=\frac{\frac{\left(m_{f}-m_{i}\right)}{{\mathrm{MW}}_{{\mathrm{CO}}_{2}}}}{m_{i}}\cdot 1000$$

In this study, the kinetics of CO_2_ adsorption onto the adsorbents were analyzed using the pseudo-first order model (PFO), pseudo-second order model (PSO), and the Avrami model, using linear and non-linear fitting. PFO, also known as the Lagergren model, is based on the assumption that the rate of occupation of adsorption sites is proportional to the number of unoccupied sites [39]. The model is expressed as

(2) $$\frac{{\mathrm{dq}}_{t}}{\mathrm{dt}}=k_{1}\left(q_{e}-\;q_{t}\right)\overset{\mathrm{Integrating}}{\to }\ln\left(q_{e}-q_{t}\right)=\ln\left(q_{e}\right)-k_{1}t$$

where q_t_ is the amount of adsorbate adsorbed at time t (mg/g), q_e_ is the equilibrium adsorption capacity (mg/g), and k_1_ is the pseudo-first order rate constant (min^−1^). A linear plot of ln(q_e_ − q_t_) versus *t* allows for the determination k_1_ and q_e_.

The PSO model assumes that the adsorption process is controlled by chemisorption involving valence force sharing or exchanges of electrons between the adsorbent and adsorbate. The model is given by [50]

(3) $$\frac{{\mathrm{dq}}_{t}}{\mathrm{dt}}={\;k}_{1}{\left(q_{e}-q_{t}\right)}^{2}\;\overset{\mathrm{Integrating}}{\to }\;\frac{t}{q_{t}}=\frac{1}{k_{2}q_{e}^{2}}+\frac{t}{q_{e}}$$

where k_2_ is the pseudo-second order rate constant (g·mg^−1^·min^−1^). A linear plot of t/q_t_ versus *t* allows for the determination of k_2_ and q_e_.

The Avrami equation, originally developed for phase transformation in material science, has been adapted to model adsorption kinetics. This equation helps to understand the kinetics of adsorption processes by describing the fraction of adsorbate uptake over time, accounting for the nucleation and growth of adsorption sites [51]. The equation is described as

(4) $$\frac{{\mathrm{dq}}_{t}}{\mathrm{dt}}=k_{A}^{n_{A}}t^{\left(n_{A}-1\right)}\left(q_{e}-q_{t}\right)\overset{\mathrm{Linearized}}{\to }\ln\left(\ln\left(\frac{q_{e}}{q_{e}-q_{t}}\right)\right)=n_{A}\ln k_{A}+n_{A}\ln t$$

where k_A_ is the Avrami kinetic constant (s^−1^) and n_A_ is the Avrami exponent, which indicates the adsorption mechanism. A linear plot of t/q_t_ versus *t* allows for the determination of k_2_ and q_e_. By fitting experimental data to these models, researchers can determine the kinetic parameters and better understand the adsorption process [52]. Only points from the adsorption phase are taken into account during the modeling process, as adding extra points from the equilibrium could lead to inaccuracies in the results [39].

An adjusted coefficient of determination was calculated according to the equation described below:

(5) $$R^{2}=1-(\frac{n-1}{n-p})\;\frac{\mathrm{SSE}}{\mathrm{SST}}$$

where SSE is the sum of squared error, SSR is the sum of squared regression, SST is the sum of squared total, n is the number of data, and p is the number of regression coefficients.

#### 3.4.2. H_2_S Removal at Low Temperature

**Figure 12:**
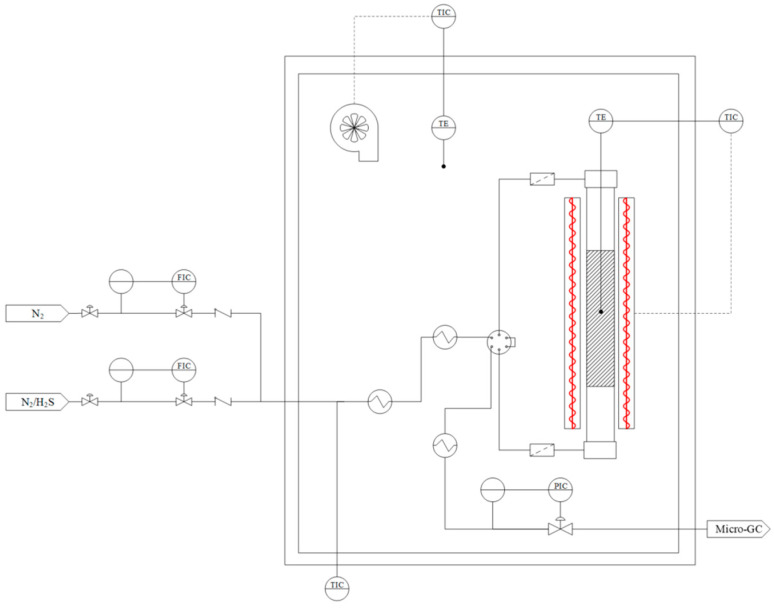
Microactivity Pro unit diagram used for the H_2_S adsorption.

Dynamic H_2_S capture studies were performed in a Microactivity Pro unit, as illustrated in Figure 12. The adsorbents are introduced into a Hastelloy C tubular reactor to prevent corrosion and issues arising from the presence of sulfur compounds. The reactor, with a diameter of 1.02 cm and a height of 380 cm, is placed in a cylindrical furnace to control the temperature. The entire system is enclosed in an electrically heated box, which can preheat the inlet gases. Gases are injected and mixed using mass flow controllers (MFCs). TIC (temperature indicator controller) and PIC (pressure indicator controller) devices regulate the system’s temperature and pressure. The maximum gas flow rate is 4.5 NL/min, and the system allows for reactions to be studied at temperatures up to 600 °C and pressures up to 30 bar. Further details are provided in previous works published by the authors and research group members [53,54].

Inlet and outlet gas streams were analyzed with a Varian 490 microGC (Palo Alto, California, USA) dual channel. The H_2_S concentration was determined using 10 m PPQ column.

Hydrogen sulfide was fed with bottled gas (100 ppmv H_2_S, nitrogen balance), and an inert atmosphere for system cleaning was provided using pure N_2_ (99.999%). A computerized system managed and controlled the different process stages. A full experiment involved the following steps: (1) system cleaning through inert gas flow (N_2_) and (2) desulfurization under process conditions at atmospheric pressure (1 bar) and 30 °C, with a weight hourly space velocity (WHSV) set at 1000 h^−1^. The tests were stopped at the breakthrough concentration of 10 ppmv. The capacities of each sorbent were calculated by the integration of the area above the breakthrough curves. To determine the effect of moisture present on the bioadsorbent, experiments were conducted in which the adsorbents were dried at 120 °C for 2 h under a N_2_ flow prior to adsorption.

## 4. Conclusions

This study demonstrates the potential of producing adsorbents for gaseous pollutant capture from pistachio shells through pyrolysis followed by biochar activation. Chemical activation with KOH significantly improves pore development, obtaining the highest adsorption capacity among the other adsorbents, with PSB-CA reaching an adsorption capacity of 2.17 mmolCO_2_/g_adsorbent_. Non-linear kinetic models are the most appropriate methods to describe CO_2_ adsorption data, with the Avrami model providing the best fit for PS-based activate biochar. For H_2_S capture, the tests showed that chemical activation yielded better results compared to physical activation. This is attributed to the higher number of active sites on the adsorbent surface, which facilitate H_2_S dissociation. PSB-CA demonstrated better performance, achieving an adsorption capacity of 9.6 mgH_2_S/g_adsorbent_ compared to 0.58 mgH_2_S/g_adsorbent_ of PSB-PA. Additionally, the water presence in the adsorbent, while it might appear to limit the pore accessibility, is required for H_2_S dissociation, thereby increasing its adsorption capacity. The hydrophilic properties of PSB-CA contribute significantly to the better results obtained in this study.

## Figures and Tables

**Table 1:** Ultimate analysis (data in percentage of the total on a dry matter basis) and properties of the adsorbents.

Adsorbent	C% (d.b)	H% (d.b)	N% (d.b)	S% (d.b)	O% ^1^ (d.b)	H/C	O/C	Ash%	pH	ρ (g/mL)	Production Yield (%)
PS	45.5	6.8	0.10	<0.10	47.7	1.8	0.79	<0.10	6.87	0.579	-
PSB	88.4	3.6	0.33	<0.10	7.42	0.49	0.06	0.25	9.11	0.407	23.3
PSB-CA	77.7	2.5	0.28	<0.10	15.72	0.39	0.15	3.8	9.4	0.479	22.8
PSB-PA	95.3	1.6	0.42	<0.10	2.36	0.20	0.02	0.32	8.84	0.449	20.2

d.b: Dry matter basis, ^1^ obtained by subtracting the sum of the CHNS and ash contents.

**Table 2:** Adsorbent surface area characterization and porosimetry values.

Adsorbent	BET Area(m^2^/g)	Area Micro (m^2^/g)	Area External (m^2^/g)	V_T_ (cm^3^/g)	V_p micro_ (cm^3^/g)	V_p meso_ (cm^3^/g)	D_Avg_ (nm)	Total Intrusion Volume (cm^3^/g)	Total Pore Area (m^2^/g)	Porosity (%)
PS	0.2	0.0004	0.22	0.001	-	0.001	6.12	0.16	4.3	17
PSB	293	238	55	0.14	0.11	0.031	1.92	0.58	9.6	47
PSB-CA	531	448	82	0.23	0.21	0.024	1.90	0.73	8.7	47
PSB-PA	340	165	174	0.16	0.07	0.08	1.82	0.67	7.2	45

**Table 4:** Adsorption kinetic parameters obtained by the linear fitting of CO_2_ on biochar derived from pistachio shell at 30 °C.

Model	Parameter	PS	PSB	PSB-PA	PSB-CA
PFO	q_e_ (mg g^−1^)	7.6	48	43	62
	k_1_ (min^−1^)	0.035	0.64	0.72	0.46
	R^2^	0.9967	0.9643	0.9435	0.9413
PSO	q_e_ (mg g^−1^)	11	83	80	115
	k_2_ (min^−1)^	0.0038	0.0017	0.0072	0.0043
	R^2^	0.9950	0.2979	0.8458	0.8505
Avrami	q_e_ (mg g^−1^)	8.7	40	57	96
	n_Av_	0.32	0.62	1.13	1.04
	k_Av_ (min^−1^)	0.052	2.20	0.75	0.56
	R^2^	0.9953	0.9794	0.9692	0.9580
Experimental	q_e exp_ (mg g^−1^)	8.4	42	57	87

**Table 5:** Adsorption kinetic parameters obtained by the non-linear fitting of CO_2_ on biochar derived from pistachio shell at 30 °C.

Model	Parameter	PS	PSB	PSB-PA	PSB-CA
PFO	q_e_ (mg g^−1^)	7.7	41	58	92
	k_1_ (min^−1^)	0.050	0.60	0.99	0.67
	R^2^	0.9970	0.9667	0.9997	0.9976
PSO	q_e_ (mg g^−1^)	10	49	71	115
	k_2_ (min^−1)^	0.0043	0.0013	0.014	0.0058
	R^2^	0.9997	0.9286	0.9692	0.98629
Avrami	q_e_	8.3	40	55	89
	N_Av_	0.86	1.64	1.29	1.12
	k_Av_ (min^−1^)	0.052	0.62	0.99	0.70
	R^2^	0.9995	0.9986	0.9997	0.9996
Experimental	q_e exp_ (mg g^−1^)	8.4	42	57	87

## Data Availability

Data available on request from the authors.

## Supplementary Materials

The following supporting information can be downloaded at https://www.mdpi.com/article/10.3390/molecules30071501/s1, Figure S1: EDS spectrum for fresh PS (A), PSB (B), PSB-CA (C) and PSB-PA (D); Figure S2: Normal probability plots of the adsorbents for the non-linear models studied; Table S1: Elemental analysis of the surface based on SEM-EDS analysis of the adsorbents for H_2_S capture; Figure S3: SEM and EDS mapping of the PSB fresh (A), PSB after H_2_S adsorption (B) and after adsorption under dry condition. Sulfur content is represented in B-II and C-II in color red; Figure S4: SEM and EDS mapping of the PSB-PA fresh (A), PSB-PA after H_2_S adsorption (B) and after adsorption under dry condition. Sulfur content is represented in B-II and C-II in color red.

## Abbreviations

The following abbreviation are used in this manuscript:

BET	Brunauer–Emmett–Teller
BJH	Barrett–Joyner–Halenda
DTG	Derivative thermogravimetry
IUPAC	International Union of Pure and Applied Chemistry
MFCs	Mass flow controllers
PFO	Pseudo first order
PIC	Pressure indicator controller
PS	Pistachio shell
PSB	Pistachio shell biochar
PSB-CA	Pistachio shell biochar chemically activated
PSB-PA	Pistachio shell biochar physically activated
PSO	Pseudo second order
SSA	Specific surface area
TGA	Thermogravimetric analysis
TIC	Temperature indicator controller
WHSV	Weight hourly space velocity
