# Supplementary material for: Development of Pistachio Shell-Based Bioadsorbents Through Pyrolysis for CO2 Capture and H2S Removal
Source: Molecules. 2025 Mar 27;30(7):1501. doi: 10.3390/molecules30071501 (PMC11990416; doi:10.3390/molecules30071501)
Supplement: Supplementary file 1 [file molecules-30-01501-s001.zip › molecules-3456766-supplementary.pdf]

Development of Pistachio Shell-Based Bioadsorbents Through Pyrolysis for CO_2 Capture and H_2S Removal

Alejandro Márquez Negro [1,2,*], Verónica Martí [1], José María Sánchez-Hervás [1] and Isabel Ortiz [1]

[1] Unit for Sustainable Thermochemical Valorization, Energy Department, CIEMAT, 28040 Madrid, Spain; veronica.marti@ciemat.es (V.M.); josemaria.sanchez@ciemat.es (J.M.S.-H.); isabel.ortiz@ciemat.es (I.O.)
[2] Department of Chemical Engineering and Materials, Faculty of Chemistry, Complutense University of Madrid (UCM), 28040 Madrid, Spain
* Correspondence: alejandro.marquez@ciemat.es

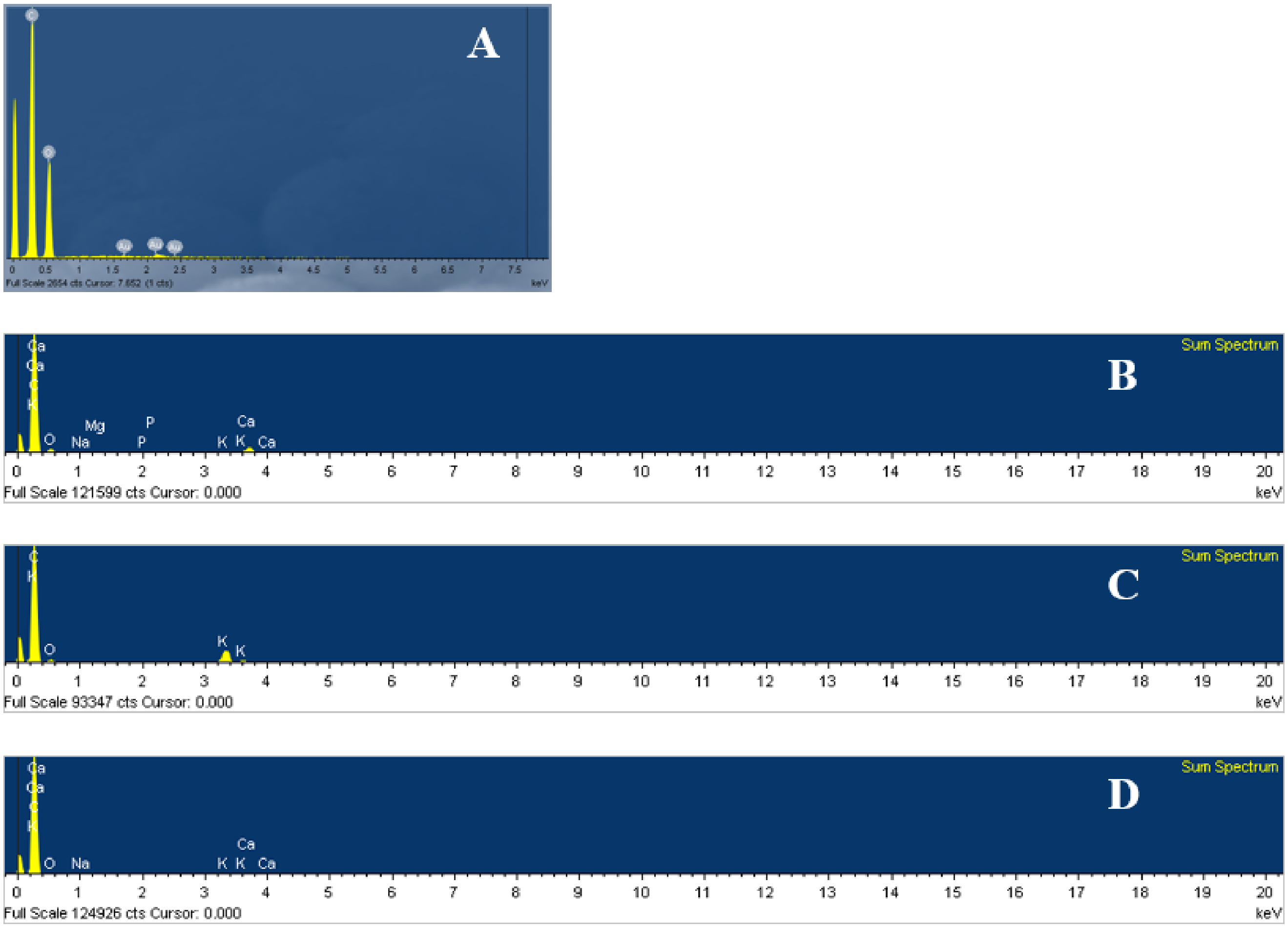

Figure S1. EDS spectrum for fresh PS (A), PSB (B), PSB-CA (C) and PSB-PA (D).

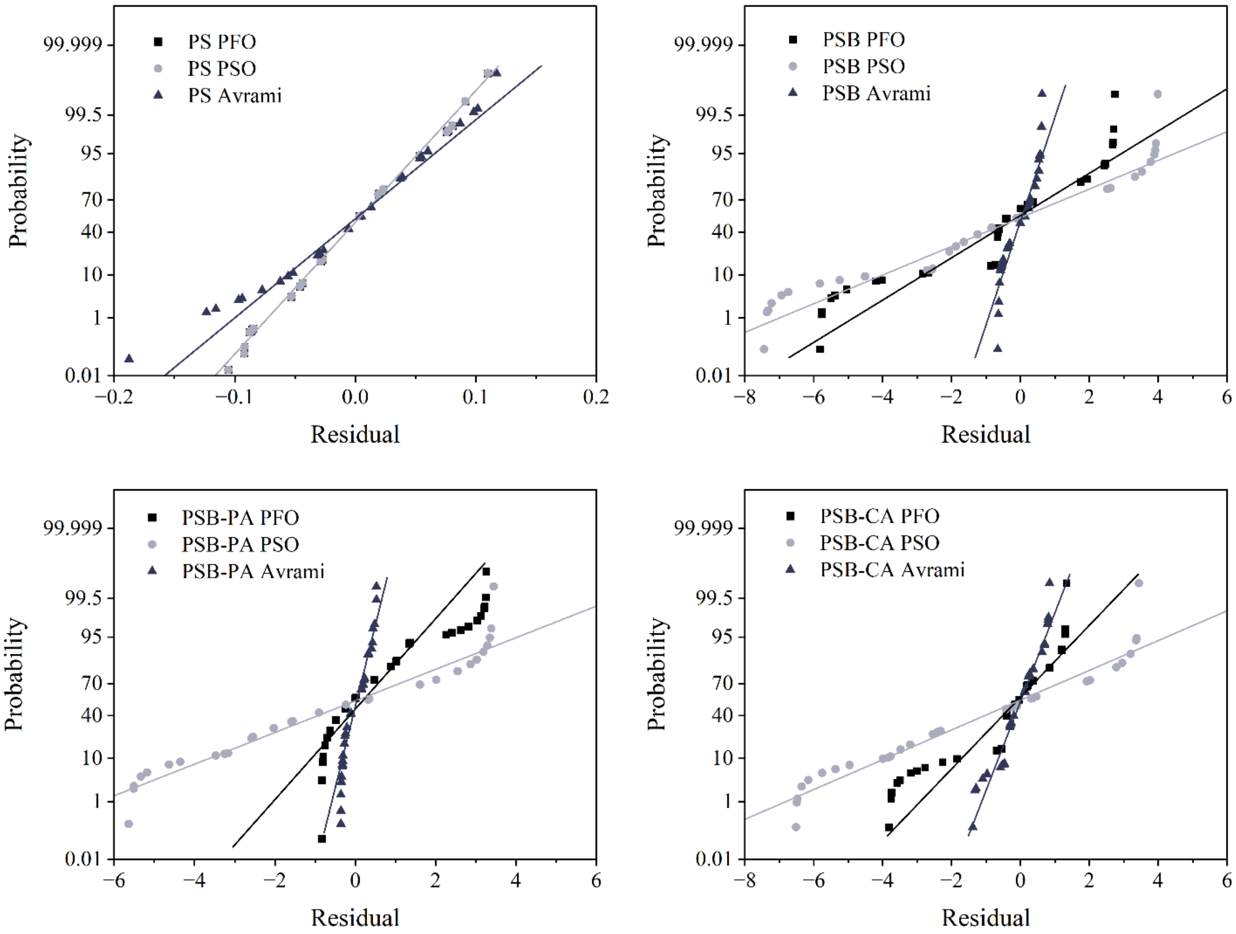

Figure S2. Normal probability plots of the adsorbents for the non-linear models studied.

Table S1. RMSE Comparison of Linear and Non-Linear Kinetic Models for the adsorbents studied.

	**Model**	**PS**	**PSB**	**PSB-CA**	**PSB-PA**
Linear fitting	PFO	0.88	6.3	20	14
	PSO	0.16	30	17	20
	Avrami model	0.37	1.0	1.4	0.57
Non-linear fitting	PFO	0.11	2.0	1.17	1.7
	PSO	0.03	2.9	2.8	2.6
	Avrami model	0.04	0.40	0.47	0.3

Table S2. Elemental analysis of the surface based on SEM-EDS analysis of the adsorbents for H_2S capture.

	Fresh			Spent					
Element	PSB	PSB-CA	PSB-PA	PSB	PSB (dry)	PSB-CA	PSB-CA (dry)	PSB-PA	PSB-PA (dry)
C (%)	61	61	75	68	71	49	32	80	75
O (%)	36	35	24	31	28	42	49	19	24
S (%)	-	-	-	0.6	0.3	3.7	2.9	0.5	0.15
K (%)	0.1	4.9	-	0.1	0.1	5.0	15.7	0.2	0.1
Na (%)	0.8	-	0.3	0.2	0.3	-	0.2	0.2	0.2
Ca (%)	2.2	-	0.6	0.2	0.7	-	-	0.3	0.6

Data in percentage of total on a dry matter basis.

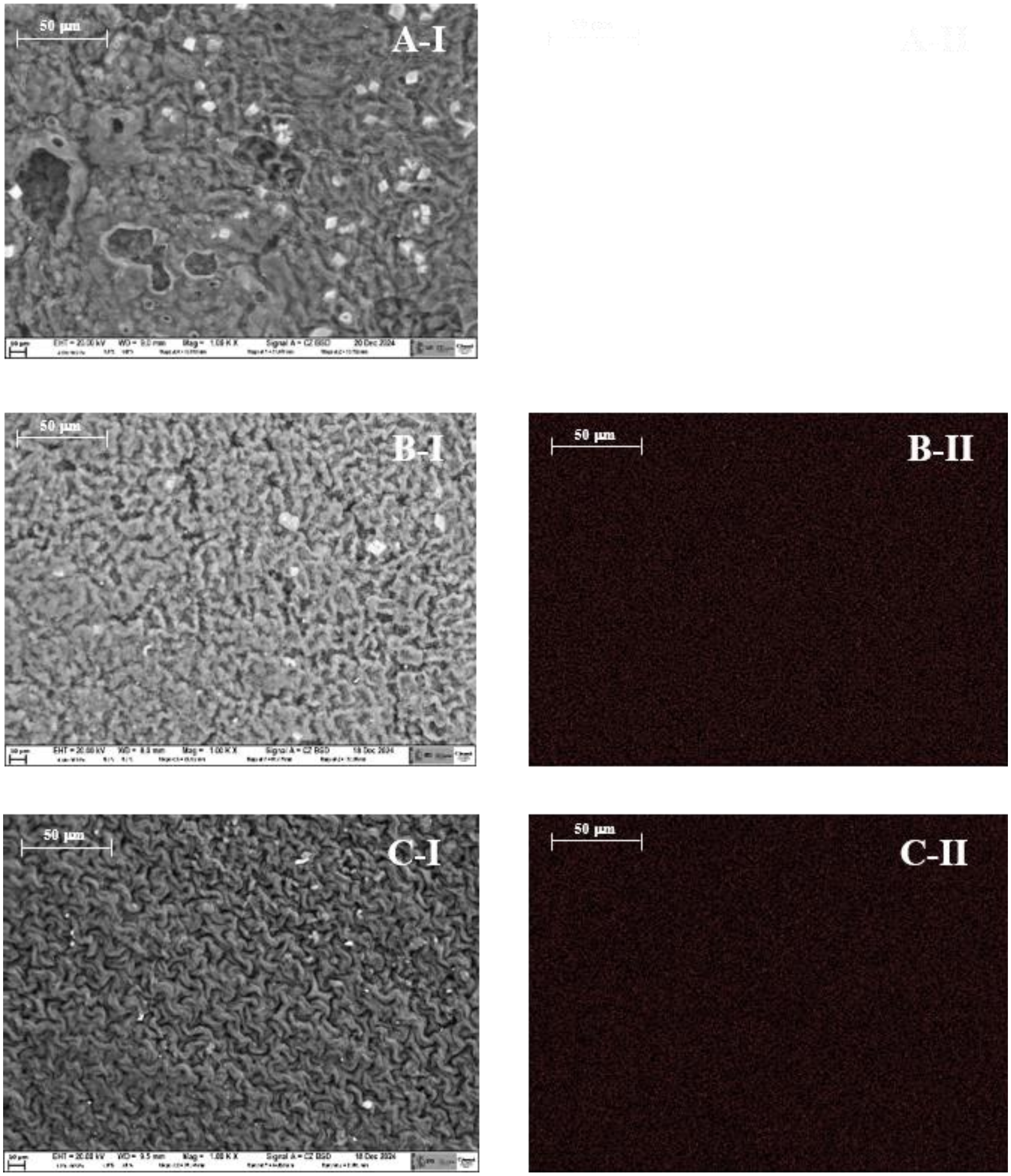

Figure S3. SEM and EDS mapping of the PSB fresh (A), PSB after H_2S adsorption (B) and after adsorption under dry condition. Sulfur content is represented in B-II and C-II in color red.

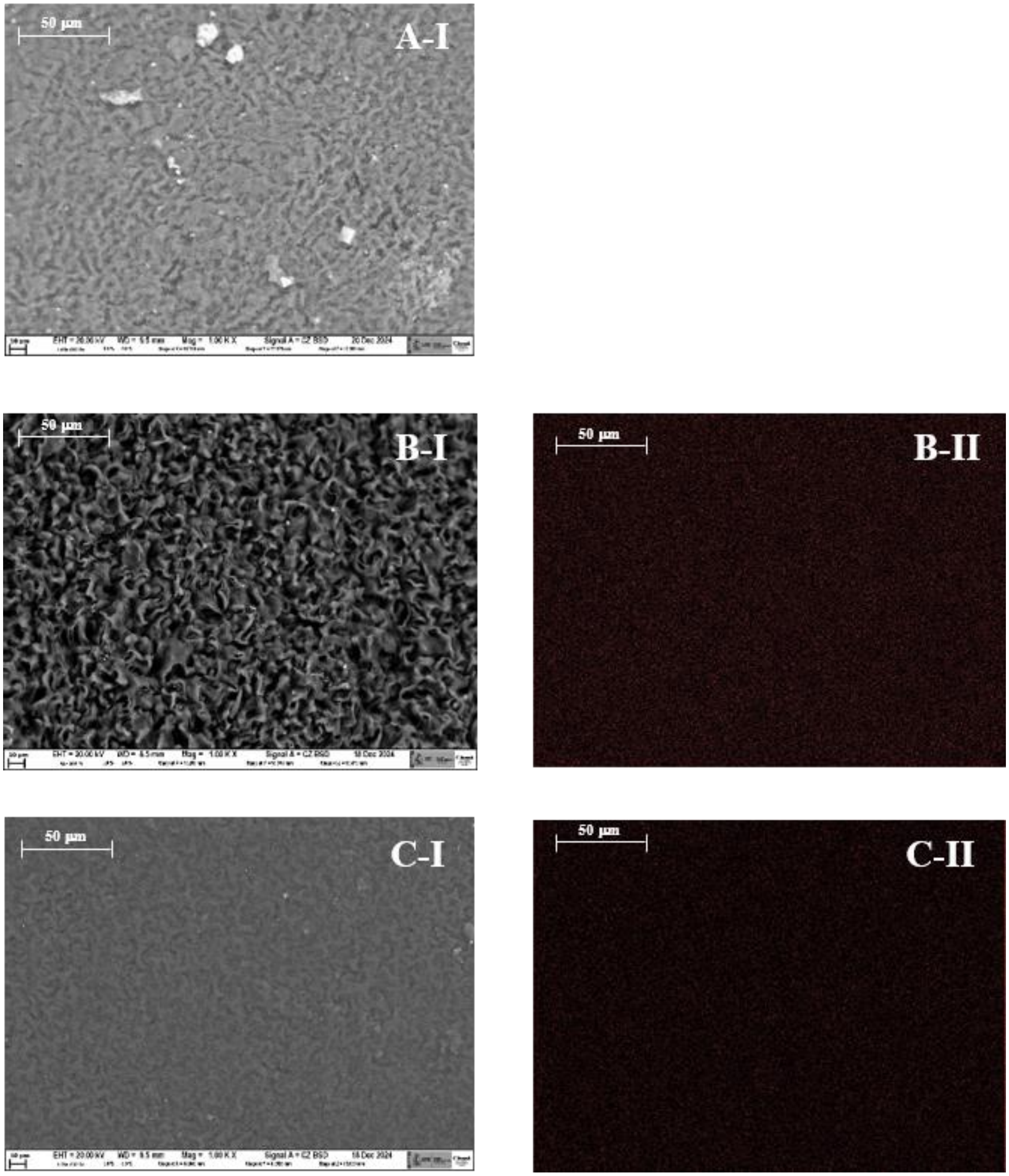

Figure S4. SEM and EDS mapping of the PSB-PA fresh (A), PSB-PA after H_2S adsorption (B) and after adsorption under dry condition. Sulfur content is represented in B-II and C-II in color red.
